# Advances in T cell–based immunotherapy for osteosarcoma

**DOI:** 10.3389/fimmu.2026.1769847

**Published:** 2026-02-05

**Authors:** Kun Zhang, Zheng Wang, Jiaqi He, Liuru Lu, Wenshu Wang, Aiwei Yang, Huayi Xie, Linhui Huang, Yuying Huang, Ke Zhang, Mingyang Jiang, Ruqiong Wei

**Affiliations:** 1 Department of Trauma Orthopedics, Shenzhen Longhua District People’s Hospital, Shenzhen, China; 2 The Second Clinical Medical College of Guangxi Medical University, Nanning, China; 3 The First Clinical Medical College of Guangxi Medical University, Nanning, China; 4 Department of Bone and Joint Surgery, The First Affiliated Hospital of Guangxi Medical University, Nanning, China; 5 Department of Rehabilitation Medicine, The First Affiliated Hospital of Guangxi Medical University, Nanning, China

**Keywords:** adaptive immunity, immune checkpoint inhibitors, immune evasion, immunotherapy, innate immunity, osteosarcoma, T cells

## Abstract

Osteosarcoma, the most prevalent primary malignant bone tumor in children and adolescents, remains a formidable clinical challenge due to its high metastatic potential and limited therapeutic progress over the past three decades. While surgery combined with multi-agent chemotherapy has improved outcomes for patients with localized disease, prognosis for those with recurrent or metastatic osteosarcoma remains poor. Although immunotherapy has revolutionized cancer care across multiple malignancies, its efficacy in osteosarcoma has been modest, largely owing to an immunosuppressive tumor microenvironment, functional T cell exhaustion, and pronounced antigenic heterogeneity. Recent advances in T cell–based strategies, including MHC-independent γδ T cells, immune checkpoint inhibitors targeting PD−1/PD−L1 and CTLA−4, and chimeric antigen receptor (CAR) T cells directed against antigens such as HER2, GD2, and B7−H3, have demonstrated encouraging preclinical activity but limited clinical translation. Emerging evidence suggests that impaired antigen presentation, suppressive immune cell populations, and inadequate T cell trafficking collectively restrict therapeutic efficacy. This review summarizes recent mechanistic and translational advances in T cell–directed immunotherapy for osteosarcoma and proposes future directions to improve clinical outcomes.

## 1 Introduction

Osteosarcoma is the most common primary malignant bone tumor, predominantly affecting children and adolescents, with a second incidence peak observed among older adults (1, 2). Current standard-of-care management, consisting of surgical resection combined with perioperative chemotherapy. However, outcomes for individuals with metastatic osteosarcoma remain dismal; published data indicate that the 5-year survival rate is below 20%, with little appreciable improvement over the past three decades (3). This stagnation highlights an urgent need to identify effective therapeutic strategies for patients with advanced-stage disease. In recent years, immunotherapy has emerged as a transformative modality across several malignancies, reshaping therapeutic paradigms by harnessing anti-tumor immune responses (4–7).

Approaches targeting T-cell–mediated immunity—such as immune checkpoint inhibitors (ICIs) and chimeric antigen receptor T-cell (CAR-T) therapies—have achieved remarkable success in selected tumor types and are considered prototype strategies for next-generation cancer treatment (8–10). Notably, osteosarcoma has been reported to harbor a substantially higher density of tumor-infiltrating T cells than many other sarcoma subtypes, suggesting an immunologically active tumor microenvironment and providing a theoretical rationale for the application of immunotherapeutic interventions (11, 12). Despite these promising observations, osteosarcoma remains largely refractory to currently available immune-based therapies, underscoring the complexity of its immune landscape and the need for deeper mechanistic understanding (13, 14). This review summarizes recent foundational and clinical advances in cell-based immunotherapy for osteosarcoma, with the aim of informing future therapeutic optimization and translational research.

## 2 The immune system and cancer immunotherapy

The immune system constitutes a highly sophisticated and integrated network, orchestrating a multitude of immune cells and molecular mediators to preserve host homeostasis (15–17). This network is fundamental for defense against exogenous pathogens and the detection of endogenous cellular aberrations, all while maintaining strict self-tolerance (18, 19). Immunological defense is classically segmented into two complementary branches: innate and adaptive immunity. The innate immune response provides an immediate, non-specific first line of defense, mediated by cells such as dendritic cells (DCs), macrophages, natural killer (NK) cells, and neutrophils (20–22). In contrast, the adaptive immune response confers antigen-specific memory and potency, primarily executed by B lymphocytes, CD4^+^ helper T cells, and CD8^+^ cytotoxic T lymphocytes (23–25). These two systems operate in concert to enable the precise recognition and elimination of invading pathogens, infected cells, and malignant transformations.

The interaction between the immune system and neoplastic development is a dynamic and multifaceted process, encompassing critical phases of immune surveillance, immune cell infiltration, and the eventual elimination of transformed cells (26–28). A principal hallmark of oncogenesis is the capacity for immune evasion, whereby malignant clones develop mechanisms to subvert immunological detection. Such mechanisms frequently involve the loss or alteration of tumor-specific antigens, rendering neoplastic cells less visible to immune effectors (29). Under physiological conditions, nascent transformed cells are typically identified and eradicated through coordinated actions, including NK cell activation, the production of interferons, and the subsequent maturation of DCs, which are essential for priming antigen-specific cytotoxic T-cell responses (30, 31). However, tumor cells frequently develop immune resistance by secreting immunosuppressive cytokines or downregulating immunogenic molecules. These adaptations effectively enable tumors to circumvent immune-mediated destruction and facilitate disease progression (32).

This complex interaction is conceptualized in the “cancer immunoediting” model, which comprises three sequential but overlapping phases: elimination, equilibrium, and escape (33, 34). During the elimination phase, immunogenic tumor antigens are identified, leading to clonal expansion of tumor-reactive T cells and targeted tumor cell destruction (34–36). In the equilibrium phase, tumor cells that evade initial clearance may persist in a dormant state under continuous immune pressure, wherein adaptive immune responses, particularly memory T cells, play a critical role in suppressing outgrowth (34, 37). Eventually, in the escape phase, subclones of tumor cells acquire further immune-resistant features and proliferate unchecked (38, 39). The downregulation of antigen-presenting machinery (HLA molecules), recruitment of immunosuppressive cell types such as M2-polarized macrophages, and upregulation of inhibitory immune checkpoints play important roles in immune escape (40–42). Notably, tumor-infiltrating lymphocytes (TILs) may become functionally impaired through increased expression of inhibitory receptors (CTLA-4, PD-1), while tumor cells may overexpress their corresponding ligands (PD-L1), leading to T cell exhaustion and immune dysfunction (43–45).

## 3 Innate cell-based therapies: γδ T cells in osteosarcoma immunotherapy

The innate immune system is distinguished by its capacity to mount anti−tumor responses in the absence of prior antigen sensitization, a process typically driven by pro−inflammatory signals such as tumor necrosis factor−α (TNF−α) and the Fas–Fas ligand axis (46, 47). Although NK cells have long been regarded as the principal effectors of innate antitumor immunity, emerging evidence underscores the pivotal contribution of γδ T cells in tumor immunosurveillance and immunotherapy (48, 49). Unlike conventional αβ T cells, which express αβ T−cell receptors (TCRs), γδ T cells carry a γδ TCR and constitute approximately 10% of the total peripheral T−cell pool (50). While many facets of their biology remain incompletely understood, γδ T cells are characterized by low antigen specificity, MHC−independent activation, and potent cytotoxic and cytokine−secreting functions (51, 52). These attributes render them particularly suited for targeting malignancies that evade adaptive immune recognition. Among human γδ T−cell subsets, the Vγ9/Vδ2 population has garnered considerable attention for its capacity to detect isopentenyl pyrophosphate (IPP), a metabolite of the mevalonate pathway, and to exert potent cytolytic activity against diverse tumor types (53, 54). Notably, synthetic phosphoantigens, when administered in combination with interleukin−2 (IL−2), induce γδ T−cell activation by promoting intracellular accumulation of IPP through inhibition of farnesyl pyrophosphate synthase (55, 56). In a clinical study of hormone−refractory prostate cancer, Francesco et al. (57) demonstrated that treatment with phosphoantigens and IL−2 led to robust activation of Vγ9/Vδ2 T cells, favorable clinical outcomes, and a well−tolerated safety profile. Furthermore, γδ T cells have been integrated into adoptive cell therapy platforms, in which autologous γδ T cells are isolated, expanded ex vivo using phosphoantigens and IL−2, and subsequently reinfused into patients. Preliminary data from several Phase I/II trials have reported encouraging safety and initial signs of antitumor efficacy (58, 59).

In osteosarcoma, research on γδ T cells remains predominantly at the preclinical stage. These cells exhibit robust anti-proliferative and cytotoxic activity against osteosarcoma cell lines, including putative cancer stem-like subpopulations (60). Recent studies indicate that combination strategies incorporating phosphoantigens and nitrogen-containing bisphosphonates—such as pamidronate or zoledronate—enhance γδ T cell–mediated cytotoxicity against osteosarcoma cells (15, 16). Beyond their MHC-unrestricted recognition and phosphoantigen responsiveness, γδ T cells deploy a diverse arsenal of effector mechanisms to mediate anti-tumor immunity. A principal cytotoxic pathway involves the granzyme B/perforin axis, whereby activated γδ T cells release perforin to form pores in target cell membranes, facilitating the entry of granzyme B and subsequent induction of apoptosis (61). This mechanism has been directly implicated in the killing of osteosarcoma cells *in vitro*, particularly after stimulation with nitrogen-containing bisphosphonates such as zoledronate, which potentiate γδ T cell cytotoxic degranulation (62). Furthermore, γδ T cells are prolific producers of pro-inflammatory cytokines, notably interferon-γ (IFN-γ) and TNF-α, both of which contribute to remodeling the tumor microenvironment and recruiting secondary immune effectors (61, 63). IFN-γ upregulates MHC class I expression on tumor cells and activates antigen-presenting cells (APCs), whereas TNF-α can promote tumor necrosis and stimulate dendritic cell maturation (64, 65). Additionally, γδ T cells engage in crosstalk with dendritic cells (DCs) and myeloid-derived suppressor cells (MDSCs) to modulate innate and adaptive immune responses. For instance, through CCL20 interactions and cytokine secretion, γδ T cells can promote DC maturation, thereby enhancing the priming of αβ T cell responses (66–68). emerging evidence suggests that γδ T cells may also counteract MDSC-mediated immunosuppression, thereby alleviating a major barrier to T cell activation in osteosarcoma (69, 70). These immunomodulatory functions position γδ T cells not merely as direct cytotoxic effectors, but as pivotal orchestrators of anti-tumor immunity within the osteosarcoma microenvironment (**Supplementary Table S1**).

## 4 Adaptive T cell–based therapies in osteosarcoma immunotherapy

### 4.1 Tumor vaccines and dendritic cell vaccines

Tumor vaccines constitute one of the earliest immunotherapeutic strategies, designed to elicit antitumor immune responses through the delivery of tumor-associated antigens (TAAs). Available vaccine platforms encompass whole−cell lysates, peptides, proteins, DNA, RNA, and synthetic long peptides (71, 72). In preclinical studies, diverse vaccine formats—including peptide− and RNA−based approaches targeting TAAs such as SART3 and WT1—have demonstrated promising immunogenicity, as evidenced by *in vitro* T−cell activation assays and murine osteosarcoma models (73, 74). Furthermore, advances in proteomic screening have facilitated the identification of potential neoantigens, thereby enhancing the prospect of antigen−specific vaccine design. Despite these preclinical advances, early clinical trials employing autologous tumor cell vaccines yielded limited therapeutic benefit in osteosarcoma patients (4, 64). A critical determinant of effective peptide vaccination is the endogenous presentation of peptide–HLA complexes on tumor cells. However, assessing such presentation has remained methodologically challenging, largely due to the scarcity of high−avidity, soluble T−cell receptors (TCRs) specific for TAAs. Recently, researchers have developed two soluble TCR multimers directed against survivin−2B (SVN−2B) and PBF peptides (HLA−A*24:02). These reagents were derived from cytotoxic T−lymphocyte clones isolated from vaccinated patients and enable the direct detection of naturally presented TAA peptides, thus highlighting the utility of TCR multimers as precision tools for advancing osteosarcoma immunotherapy (75).

DCs are professional APCs capable of activating T cells and promoting cytotoxic T lymphocyte expansion (76, 77). First-generation DC vaccines are typically produced by isolating peripheral blood mononuclear cells or hematopoietic progenitors from patients, differentiating them *in vitro* with GM-CSF, pulsing with tumor antigens, and then reinfusing the mature DCs back into the host. Clinical studies have confirmed the safety and immunogenicity of this approach (78). In immunocompetent murine models, DC vaccines loaded with osteosarcoma lysates or tumor−derived RNA have demonstrated an ability to impede tumor progression and promote antigen−specific T cell activation, thereby indicating a capacity for immunological tumor control. Nevertheless, early−phase clinical trials in human osteosarcoma patients—for example, those employing autologous tumor lysate−pulsed DCs—have to date elicited only modest immune responses and limited clinical benefit (79, 80). These findings highlight the translational gap between experimental promise and clinical efficacy. To address these limitations, ongoing clinical investigations are focusing on combinatorial regimens, incorporating either immunostimulatory adjuvants or agents that target immunosuppressive elements within the tumor microenvironment, such as MDSCs.

### 4.2 Chimeric antigen receptor T cells

Adoptive cellular therapy employing CAR-T cells has emerged as a powerful immunotherapeutic strategy to circumvent central mechanisms of tumor immune evasion. This approach is particularly salient in contexts where tumor cells downregulate human leukocyte antigen (HLA) molecules or exhibit low intrinsic immunogenicity (81, 82). By genetically reprogramming T cells to express synthetic receptors that recognize tumor-associated antigens (TAAs), CAR-T cells mediate HLA-independent target recognition and confer direct, potent cytotoxic activity. The canonical CAR architecture comprises an extracellular single-chain variable fragment (scFv) derived from tumor-specific monoclonal antibodies, a hinge region for flexibility, and intracellular signaling motifs critical for T cell activation (83–85). First-generation CARs incorporated the CD3ζ signaling domain alone. Subsequent iterations have enhanced persistence and efficacy: second- and third-generation constructs integrate one or more costimulatory domains (CD28, 4-1BB), while fourth-generation “armored” CARs further incorporate inducible cytokine expression cassettes (IL-12) to augment antitumor function within the immunosuppressive tumor microenvironment (86). To mitigate antigen escape, dual-targeting CAR-T cells capable of recognizing multiple antigens are also under active preclinical and clinical investigation (87). Antigen selection is a key determinant of CAR-T cell efficacy. Notably, CD19-directed CAR-T therapy has achieved regulatory approval as a first salvage therapy for refractory large B-cell lymphoma, validating the clinical potential of this platform (88). HER2, a well-established oncogene in breast cancer, is frequently overexpressed in osteosarcoma, although its prognostic significance in bone tumors remains controversial. Preclinical evidence indicates that HER2-targeted CAR-T cells can effectively recognize and lyse osteosarcoma cells, even those exhibiting low levels of HER2 expression, supporting its continued investigation as a therapeutic target in this malignancy (89).

**Figure 1:**
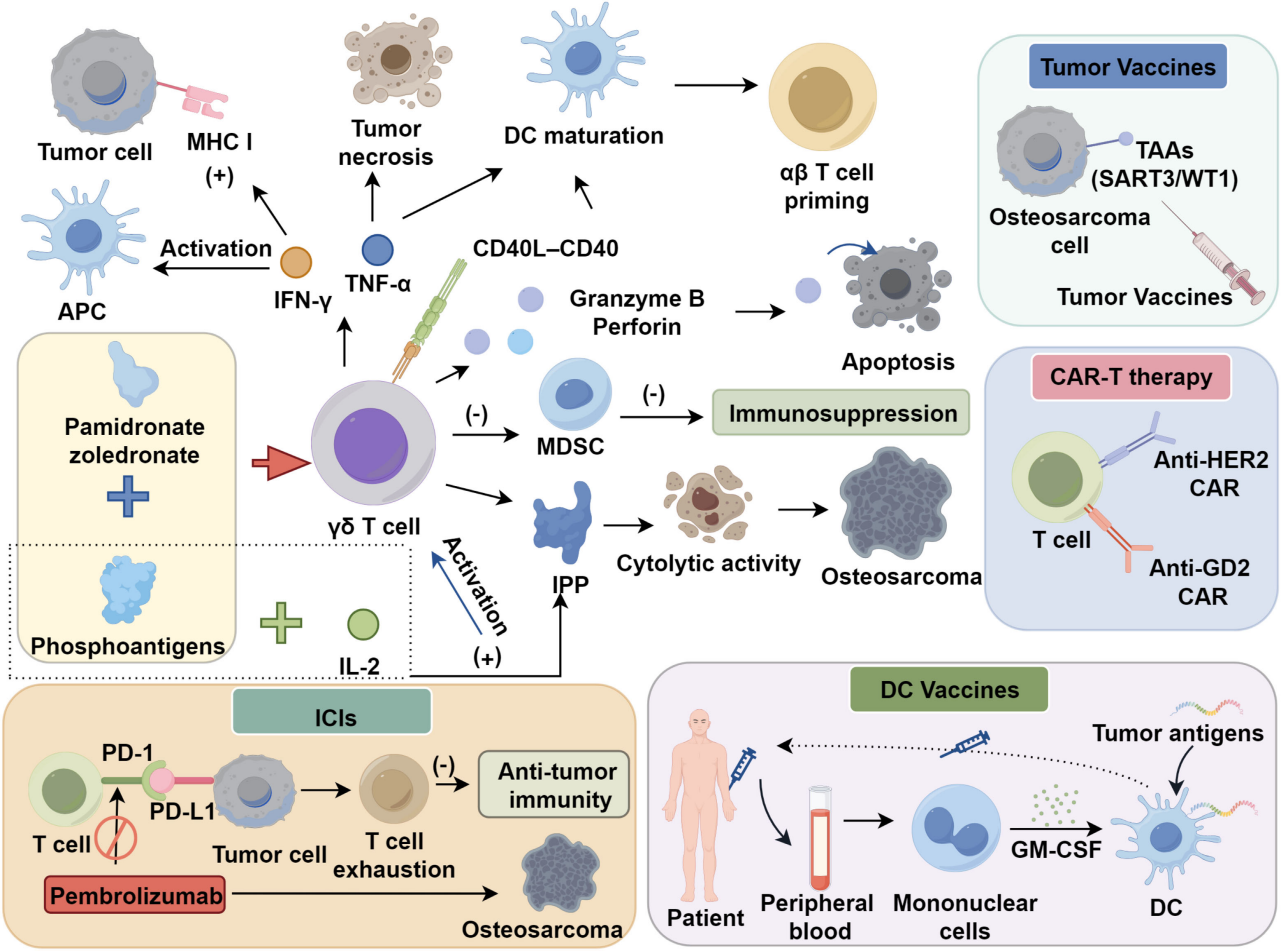
Advances in T cell–based immunotherapy for osteosarcoma.

Ahmed and colleagues (90) reported a Phase I clinical trial evaluating second−generation HER2−targeting CAR−T cells in patients with refractory HER2−positive malignancies, including osteosarcoma. Infusion−related reactions were limited to mild adverse events, and stable disease was achieved in three individuals for durations of 12–15 weeks, suggesting preliminary evidence of clinical activity. With continued refinement in CAR architecture and the identification of novel target antigens, multiple ongoing trials are assessing additional candidates for osteosarcoma, such as GD2, CD22, IL11RA, IGF−1R, ROR1, and B7−H3. Current GD2−directed CAR−T studies are further augmented by complementary engineering strategies—for example, the co−expression of chemokine receptors such as CXCR2 to potentiate T−cell trafficking and tumor infiltration (91–93). However, tumor antigen heterogeneity frequently drives the emergence of antigen−escape variants and subsequent relapse. To broaden antigen coverage and reduce the risk of immune evasion, dual−antigen CAR systems (simultaneous targeting of CD33 and CD3) and incorporation of the co−stimulatory molecule B7−H3 (CD276) have been developed (94–96). Besides, CAR−T cell exhaustion—characterized by loss of effector function and persistent upregulation of inhibitory receptors including PD−1, TIM−3, and LAG−3—is aggravated by the immunosuppressive tumor microenvironment (97, 98). Recent approaches such as incorporating checkpoint blockade (PD-1), metabolic reprogramming, or memory stem T cell–enriched formulations aim to preserve CAR-T cell persistence and cytotoxicity in hostile microenvironments (99–102). These strategies underscore the need for integrated CAR-T cell designs that address both the physical and immunologic barriers within solid tumors (**Figure 1**).

## 5 ICIs and osteosarcoma immunotherapy

To preserve immune homeostasis and avert excessive inflammation, the immune system depends on intrinsic immunoregulatory pathways. Central to this regulatory framework are immune checkpoints, which are critical for maintaining self-tolerance, preventing autoimmune pathology, and modulating immune responses. Notably, these same pathways are co−opted by malignant tumors to evade immune surveillance and establish a state of immunoresistance (103, 104). Prominent among these checkpoint molecules are CTLA−4, PD−1, and TIM−3 (105). Engagement of these receptors on T cells with their respective ligands transduces downstream signals that suppress T−cell activation and effector functions, thereby enabling tumor cells to persist undetected (106). Under physiological conditions, such checkpoint signaling is indispensable for regulating immunosuppressive cell populations, including Tregs and MDSCs (107–109). Within the tumor microenvironment, however, malignant cells exploit these mechanisms to attenuate anti−tumor immunity. Tumors secrete chemokines such as CCL20 to recruit Tregs and upregulate checkpoint ligands such as PD−L1 to directly inhibit cytotoxic T−cell responses (110, 111). PD−L1 is broadly expressed on tumor−associated macrophages and tumor cells; its interaction with PD−1 induces T−cell exhaustion, thereby impairing effective anti−tumor immunity (112, 113). Targeting this axis has yielded substantial clinical success across multiple malignancies. Anti−PD−1 agents (nivolumab) and anti−CTLA−4 agents (ipilimumab) have demonstrated durable responses in melanoma, non−small cell lung cancer, and renal cell carcinoma, frequently surpassing outcomes achieved with conventional chemotherapy (114–117).

In osteosarcoma, PD-L1 is frequently overexpressed, and its expression has been consistently correlated with unfavorable clinical prognosis (118). Preclinical investigations employing PD-1 blockade in murine models of osteosarcoma have demonstrated significant inhibition of tumor progression and an overall improvement in survival (119). Despite these promising preclinical findings, clinical outcomes in human trials have been largely discouraging. The Phase II SARC028 trial, which evaluated the PD-1 inhibitor pembrolizumab in patients with advanced osteosarcoma, reported only limited therapeutic activity (40). Similarly, another Phase II study (PEMBROSARC) combining pembrolizumab with low-dose cyclophosphamide in refractory sarcoma patients, including those with osteosarcoma, failed to demonstrate a significant improvement in objective response rate (ORR) (120). These disappointing clinical results may be attributable, in part, to the profoundly immunosuppressive characteristics of the osteosarcoma tumor microenvironment (4). Notably, osteosarcoma lesions are commonly infiltrated by abundant immunoregulatory cell populations, such as FoxP3^+^ Tregs, which actively suppress cytotoxic T lymphocyte function and thereby attenuate antitumor immunity (121, 122). Furthermore, tumor-associated macrophages within osteosarcoma tissues frequently exhibit an M2-like polarization state, characterized by expression of CD163 and CD206 and secretion of anti-inflammatory cytokines including IL-10 and TGF-β, which collectively reinforce local immune suppression (123–125). Additionally, osteosarcoma cells often downregulate major histocompatibility complex class I (MHC I) molecules, a mechanism that impairs antigen presentation to T cells and consequently diminishes the efficacy of T cell–mediated cytotoxicity (126).

## 6 Conclusion

Immunotherapy represents a paradigm shift in oncology, offering new hope for cancers previously deemed unresponsive to conventional treatment. In osteosarcoma, however, translating immunological breakthroughs into clinical benefit remains a formidable task. Accumulating evidence highlights the complex interplay between tumor cells and the immune system, with innate immune subsets like γδ T cells and adaptive platforms such as tumor vaccines, CAR-T cells, and dendritic cell therapies—each showing promise, though most remain in early investigational stages. Immune checkpoint blockade, while transformative in other solid tumors, has shown only modest benefit in osteosarcoma, underscoring the need to better understand and overcome the immunosuppressive and antigenically heterogeneous tumor microenvironment.

Future research must prioritize the identification of robust osteosarcoma-specific immune targets, refine patient stratification strategies through immunogenomic profiling, and develop rational combination therapies that synergize immune activation with other modalities such as chemotherapy, radiotherapy, or metabolic intervention. In particular, personalized neoantigen vaccines tailored to the mutational landscape of individual patients may enhance antigen specificity and immunogenicity. Bispecific T cell engagers (BiTEs) represent a promising modality to redirect cytotoxic T cells toward osteosarcoma cells with high precision. The development of armored CAR-T cells, engineered to overcome immunosuppressive barriers via the secretion of stimulatory cytokines or checkpoint blockade molecules, may further enhance therapeutic efficacy in otherwise refractory tumors. Moreover, the integration of single-cell and spatial transcriptomics is expected to refine our understanding of the osteosarcoma immune microenvironment, enabling the identification of resistant cellular subpopulations and uncovering context-specific targets for immunotherapy. Longitudinal studies are needed to elucidate the mechanisms of immune resistance and T cell dysfunction in osteosarcoma. Ultimately, advancing osteosarcoma immunotherapy will require not only technological innovation but also a nuanced appreciation of the tumor–immune interface. With strategic integration of multi-omics approaches, biomarker discovery, and translational immunology, the next generation of immunotherapy may transform the treatment landscape for this intractable malignancy.
